# SHP2 inhibitor PHPS1 ameliorates acute kidney injury by Erk1/2-STAT3 signaling in a combined murine hemorrhage followed by septic challenge model

**DOI:** 10.1186/s10020-020-00210-1

**Published:** 2020-09-21

**Authors:** Jihong Jiang, Baoji Hu, Chun-Shiang Chung, Yaping Chen, Yunhe Zhang, Elizabeth W. Tindal, Jinbao Li, Alfred Ayala

**Affiliations:** 1 Department of Anesthesiology, Shanghai General Hospital, Shanghai Jiao Tong University School of Medicine, Shanghai, 200080 P.R. China; 2 Department of Anesthesiology, Shanghai Pudong Hospital, Fudan University-Pudong Medical Center, Shanghai, 200433 P.R. China; 3 Division of Surgical Research, Department of Surgery, Aldrich 227, Rhode Island Hospital/ the Alpert School of Medicine at Brown University, 593 Eddy Street, Providence, RI 02903 USA; 4 Department of Emergency Medicine, Shanghai East Hospital, Tongji University School of Medicine, Shanghai, 200120 P.R. China

**Keywords:** SHP2, Acute kidney injury, PHPS1, Erk1/2, STAT3, Phosphatase inhibition, AKI, Hemorrhage, Shock, Sepsis

## Abstract

**Background:**

Hypovolemic shock and septic challenge are two major causes of acute kidney injury (AKI) in the clinic setting. Src homology 2 domain-containing phosphatase 2 (SHP2) is one of the major protein phosphatase tyrosine phosphatase (PTPs), which play a significant role in maintaining immunological homeostasis by regulating many facets of immune cell signaling. In this study, we explored whether SHP2 signaling contributed to development of AKI sequential hemorrhage (Hem) and cecal ligation and puncture (CLP) and whether inactivation of SHP2 through administration of its selective inhibitor, phenylhydrazonopyrazolone sulfonate 1 (PHPS1), attenuated this injury.

**Methods:**

Male C57BL/6 mice were subjected to Hem (a “priming” insult) followed by CLP or sham-Hem plus sham-CLP (S/S) as controls. Samples of blood and kidney were harvested at 24 h post CLP. The expression of neutrophil gelatinase-associated lipocalin (NGAL), high mobility group box 1 (HMGB1), caspase3 as well as SHP2:phospho-SHP2, extracellular-regulated kinase (Erk1/2): phospho-Erk1/2, and signal transducer and activator of transcription 3 (STAT3):phospho-STAT3 protein in kidney tissues were detected by Western blotting. The levels of creatinine (Cre) and blood urea nitrogen (BUN) in serum were measured according to the manufacturer’s instructions. Blood inflammatory cytokine/chemokine levels were detected by ELISA.

**Results:**

We found that indices of kidney injury, including levels of BUN, Cre and NGAL as well as histopathologic changes, were significantly increased after Hem/CLP in comparison with that in the S/S group. Furthermore, Hem/CLP resulted in elevated serum levels of inflammatory cytokines/chemokines, and induced increased levels of HMGB1, SHP2:phospho-SHP2, Erk1/2:phospho-Erk1/2, and STAT3:phospho-STAT3 protein expression in the kidney. Treatment with PHPS1 markedly attenuated these Hem/CLP-induced changes.

**Conclusions:**

In conclusion, our data indicate that SHP2 inhibition attenuates AKI induced by our double-hit/sequential insult model of Hem/CLP and that this protective action may be attributable to its ability to mitigate activation of the Erk1/2 and STAT3 signaling pathway. We believe this is a potentially important finding with clinical implications warranting further investigation.

## Introduction

Trauma patients often experience hypovolemic shock and sepsis simultaneously, resulting in severe organ dysfunction (Karasu et al. 2019; Spahn et al. 2019). The kidneys are one of the most commonly affected organs, since they receive 20–25% of the resting cardiac output, despite making up less than 1% of the total body mass. This combination makes them more vulnerable and susceptible to acute injury following blood volume losses and the fluid shifts associated with sepsis. While a better understanding of the pathogenesis of acute kidney injury (AKI) has allowed for early diagnosis and identification of high risk patients (Honore et al. 2015), the AKI-associated mortality rates remain unacceptably high (Barbar et al. 2018; Uchino et al. 2005; White et al. 2013). As a result of this, it remains critically important to further our understanding of the pathological mechanism so that we may identify novel therapeutic targets to prevent and treat AKI in those who are critically ill or severely injured.

The pathophysiology of AKI is thought to occur due to either ischemic and/or septic events that lead to metabolic derangments (Dellepiane et al. 2016). It is hypothesized that this process stems from a fundamental imbalance between pro- and anti-inflammatory processes occurring systemically (Poston and Koyner 2019). Prior studies have shown that exposure to pro-inflammatory cytokines may lead to renal cell death and dysfunction (Gomez et al. 2014). Interestingly, administration of antibiotics in an effort to treat an underlying septic process has not been shown to reduce the mortality associated with AKI in critically-ill patients (Kellum et al. 2019). Our prior research as well as that of others has shown that immune co-inhibitory receptor molecules play a role in the pathological inflammatory response which occurs in animals after hemorrhagic shock and sepsis (Patil et al. 2017; Wakeley et al. 2020). It has been shown that the suppression of T-cell function results from the recruitment of phosphatase protein tyrosine phosphatase (PTP) family members, likely due to the fact that co-inhibitory receptors from the Programmed Cell Death Receptor-1 (PD-1) family bear intracellular tyrosine inhibitory motifs (ITIM) and/or intracellular tyrosine switch motifs (ITSM) (Keir et al. 2008; Riley 2009).

Src homology 2 domain-containing phosphatase 2 (SHP2) is one of the immune system’s major PTPs. As such, it plays a significant role in maintaining immunological homeostasis by regulating multiple aspects of immune cell signaling. Chichger et al. found that SHP2 activation protects against injury to the endothelial barrier in the lung (Chichger et al. 2015). We recently reported that SHP1, but not SHP2, reduces PD-L1-dependent regulation of T regulatory lymphocyte (Treg) function and is associated with resolution of shock- and sepsis-induced lung injury (Tang et al. 2015). Additionally, previous research has demonstrated that SHP2 deficiency may have a protective effect in experimental renal (Hsu et al. 2017; Teng et al. 2018; Verma et al. 2016) and cardiovascular (Chen et al. 2018) injury. Thus, it appears that SHP2 activation may have differential effects depending on the location of action. Activation of SHP2 positively modulates the activity of extracellular-regulated kinase (Erk), which is produced following the induction of multiple cytokines (Maroun et al. 2000). In addition to its role in Grb-2–associated binder 1 (Gab1)-mediated Erk activation, SHP2 attenuates epidermal growth factor (EGF)-dependent phosphatidylinositol 3 kinase (PI3K) activation by dephosphorylating Gab1 at the p85 binding sites (Zhang et al. 2002). Given that SHP2 activity is believed to be dependent on Erk signaling, our study sets out to examine the hypothesis that the development of AKI following sequential exposure to hemorrhagic shock and sepsis, is mediated in part through dysregulated SHP2 activation resulting in stimulation of Erk1/2 as well as signal transducers and activators of transcription 3 (STAT3).

## Materials and methods

### Animals and groups

All experiments were performed in accordance with National Institutes of Health guidelines and approved by the Animal Use Committee of Rhode Island Hospital (AWC# 5064–18). A total of 18 male C57BL/6 mice (10 to 12 weeks old) were included in the experiment. The animals were maintained in a 12/12-h light/dark cycle at ambient temperature (23–25 °C) and provided with standard laboratory rodent chow and water ad libitum. Of note, the choice of male mice was made so as to maximize our ability to initially see an experimental difference in the organ injury response based on previous reports that male mice did poorer in response to these experimental stressors of shock/hemorrhage (Hem) and/or septic (CLP) challenge than pro-estrus stratified female (Wichmann et al. 1997; Zellweger et al. 1997).

Mice were randomly divided into three groups of 6 animals each: 1) the control group where mice underwent sham Hem and sham CLP procedures; 2) the Hem/CLP group; and 3) the Hem/CLP + PHPS1 (inhibitor of SHP2) group.

### Experimental protocol

Hemorrhage and sepsis were elicited as described previously in our laboratory (Biron et al. 2018). In brief, bilateral femoral arteries were catheterized under isoflurane anesthesia, allowing for continuous monitoring of the mean arterial blood pressure (MAP) using a blood pressure analyzer (MicroMed, Louisville, Ky) as well as removal of 30 to 40 % of their total estimated blood volume in order to achieve a MAP of 35 +/− 5 mmHg. Hypotension was maintained for 90 min prior to resuscitation with Ringer’s solution equivalent to four times the previously drawn blood volume. Sham mice were also anesthetized but underwent bilateral femoral artery ligation (all incisions bathed in lidocaine) only without any loss of blood volume.

Twenty four hours after Hem, mice underwent CLP technique as described previously (Biron et al. 2018). Briefly, mice were anesthetized by isoflurane and a midline abdominal incision was made. The cecum was mobilized and half of its length below the ileocecal valve was ligated, punctured twice using a 22-gauge needle with production of a small amount of feculent material in order to rapidly induce polymicrobial peritonitis. The abdominal wall was then closed in 2 layers and bathed in lidocaine. Sham CLP mice underwent a midline laparotomy, extraction and manipulation of the cecum without ligation or needle perforation. In both cases, after surgery, the mice were resuscitated by a subcutaneous injection of pre-warmed (37 °C) 0.6 mL normal saline.

In accordance with the prior study performed by Chen et al., the SHP2-selective inhibitor, phenylhydrazonopyrazolone sulfonate 1 (PHPS1) (Cayman Chemical, Ann Arbor, MI), was administered at 3 mg per kilogram body weight dissolved in DMSO/PBS in a 1:1 ratio by a subcutaneous injection once immediately after Hem and, once again, following the performance of CLP. Sham Hem/Sham CLP (S/S) mice also received the same treatment of vehicle or PHPS1.

### Determination of serum biochemical indicators

Blood urea nitrogen (BUN) and creatinine (Cre) concentrations were measured using corresponding kits (Abcam, Cambridge, MA). Blood inflammatory cytokine/chemokine levels were detected by commercial ELISA kit (BD Biosciences, San Jose, CA). All procedures were performed according to the manufacturer’s instructions.

### Calculation of kidney/body weight index (KI)

The left kidney was harvested, weighed, frozen rapidly in liquid nitrogen and stored at − 80 °C for subsequent analysis. The kidney/body weight index (KI) was calculated using the following equation: kidney wet weight (mg)/total body weight prior Sham or Hem procedure (g) × 100%. Under normal conditions, KI is relatively constant. However, in kidneys experiencing congestion, edema or cellular hypertrophy, the KI usually increases (Zhang et al. 2019).

### Renal histology

The right kidney was fixed in 4% formaldehyde, embedded in paraffin and cut into 5-μm thick sections. Hematoxylin and eosin (H&E)-stained sections were scored in a blinded, semiquantitative manner using a 0–4 scale (Wu et al. 2019). Tubular damage such as tubular necrosis, dilatation, apoptosis, and cast formation was scored as follows: 0 (none), 1 (1–10%), 2 (11–25%), 3 (26–45%), and 4 (46–75%).

### Western blot analysis

Whole tissue kidney protein lysates (30 μg total protein in each lane) were subjected to sodium dodecyl sulphate-polyacrylamide gel electrophoresis using a 10% gel and then transblotted onto polyvinylidene fluoride membranes. The protein levels of neutrophil gelatinase-associated lipocalin (NGAL), high mobility group protein B1 (HMGB1), SHP2, phospho-SHP2 (Tyr580), Erk1/2, phospho-Erk1/2, STAT3 and phospho-STAT3 and caspase 3 were determined using specific antibodies (1:1000; Cell Signaling, Danvers, MA). Glyceraldehyde 3-phosphate dehydrogenase (GAPDH) or beta-actin (1:3000; Abcam, Cambridge, USA) were used as loading control, and the amount of protein in the blots was quantified using a Bio-Rad ChemiDoc Imaging System and Image Lab 6.0 software (Bio-Rad Laboratories, California, USA).

### Statistical analysis

Values are expressed as a mean ± standard deviation. The statistical analysis was performed with the SPSS version 24.0 statistical software package (IBM Inc., Armonk, NY, USA). Data were tested for normality and equality of variance. Comparisons among the three groups for each dependent variable were performed using an analysis of variance (ANOVA) with a post hoc Newman-Keuls multiple comparison test. The level of statistical significance was set at *p* < 0.05.

## Results

### Hem/CLP-induced SHP2 activation in the kidney is attenuated by PHPS1 treatment

**Fig. 1:**
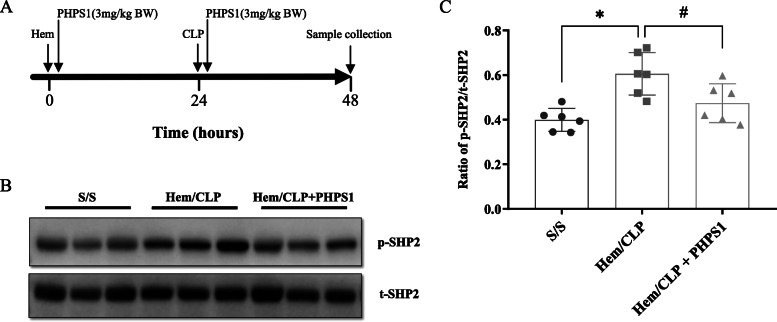
SHP2 activation in the kidney is reduced by PHPS1 treatment. Diagram of experimental timeline for PHPS1 (3 mg/kg BW) administration, Hem, CLP and mice sacrifice (**a**). Twenty four h post-Hem/CLP or Sham operation, the kidneys were collected and tissue homogenates were obtained. The extent of SHP2 activation (phosphorylation) was determined by western blot (**b**, **c**). The ratio of phosphorylated SHP2 and total SHP2 (p-SHP2/t-SHP2) in kidneys from Hem/CLP mice was significantly increased compared to Shams. PHPS1 treated Hem/CLP mice showed reduced SHP2 activation compared to Veh treated Hem/CLP mice. *, *P* < 0.05, versus Sham (S/S); #, *P* < 0.05, PHPS1 treated group (Hem/CLP + PHPS1) versus Hem/CLP + vehicle (Hem/CLP). One-way ANOVA and a Student-Newman-Keuls’ test, Mean ± SD; *n* = 6 mice/group

PHPS1, a cell-permeable highly selective inhibitor for SHP2, has been shown to inhibit SHP2-dependent cellular signaling and tumor cell colony formation (Hellmuth et al. 2008). In our study, subcutaneous injection of PHPS1 immediately after both Hem and CLP procedures resulted in a significant decrease in the level of activated SHP2 as shown in Fig. 1.

### Treatment with PHPS1 reduces the extent of Hem/CLP-induced kidney injury

**Fig. 2:**
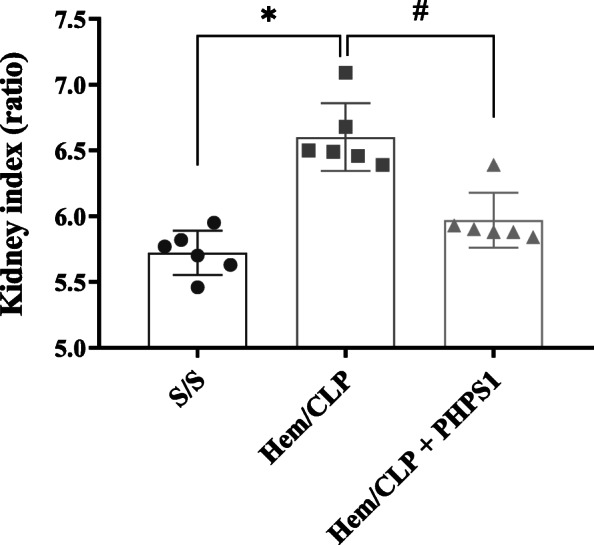
PHPS1 treatment decreased kidney/body weight index (KI) elevated by Hem/CLP. The kidneys were collected 24 h post Hem/CLP or Sham operation. Vehicle treated Hem/CLP mice showed increased KI compared to Shams and PHPS1 treated Hem/CLP mice. *, *P* < 0.05, versus Sham (S/S); #, *P* < 0.05, PHPS1 treated group (Hem/CLP + PHPS1) versus Hem/CLP + vehicle (Hem/CLP). One-way ANOVA and a Student-Newman-Keuls’ test, Mean ± SD; *n* = 6 mice/group

We observed a marked increase in the KI of Hem/CLP mice. This is important since an increased KI correlates with kidney injury such as edema, hypertrophy and organ congestion. While the KI in Hem/CLP group mice increased compared with the sham group, this change was suppressed with PHPS1 treatment as shown in Fig. 2. This implies that PHPS1 treatment directly or indirectly reduced the kidney edema and cell hypertrophy occurring here.

### SHP2 inhibition provides a renal protective effect in Hem/CLP mice

**Fig. 3:**
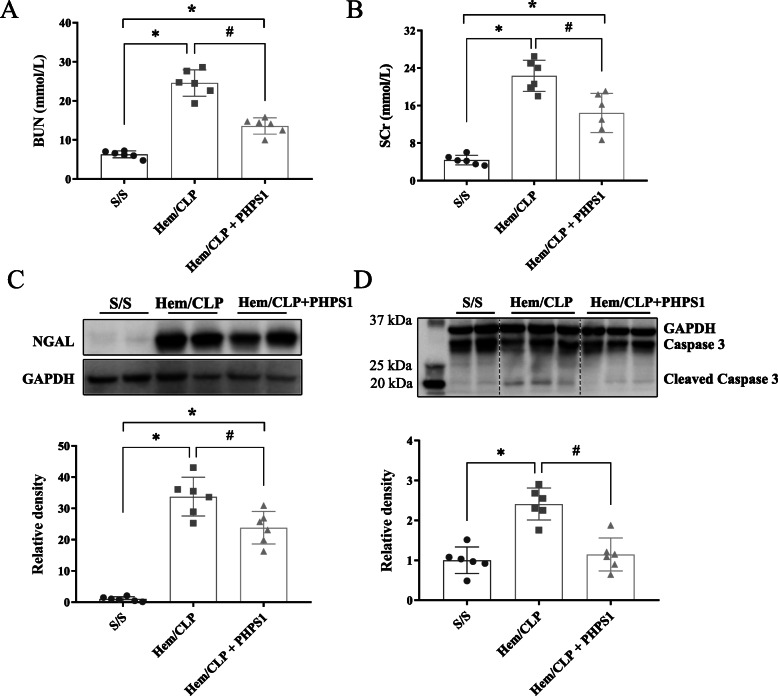
Effects of SHP2 inhibition by PHPS1 treatment on kidney injury after Hem/CLP. The plasm samples were collected 24 h post Hem/CLP or Sham operation for urea nitrogen (BUN) (**a**), creatinine (SCr) (**b**), NGAL (**c**) and cleaved caspase 3 (**d**). Vehicle treated Hem/CLP mice showed significantly increased in BUN, SCr, NGAL and cleaved caspase 3 levels compared to Sham mice. However, these increases were markedly reduced in PHPS1 treated Hem/CLP mice. *, *P* < 0.05, versus Sham (S/S); #, *P* < 0.05, PHPS1 treated group (Hem/CLP + PHPS1) versus Hem/CLP + vehicle (Hem/CLP). One-way ANOVA and a Student-Newman-Keuls’ test, Mean ± SD; *n* = 6 mice/group

As expected, mice subjected to Hem/CLP exhibited significantly elevated serum BUN and Cre levels when compared to that of sham mice. We once again observed that treatment with PHPS1 significantly improved renal function as evidenced by reduction in the levels of BUN and Cre (Fig. 3a and b).

It has been reported that NGAL is a promising biomarker for diagnosis of AKI (Kellum et al.) and we used Western Blot to detect changes in expression levels. As shown in Fig. 3c, NGAL was significantly upregulated in renal tissue following Hem/CLP when compared with sham mice. We once again found that administration of PHPS1 reversed this trend. Expression of cleaved or activated caspase 3 in renal tissue was also examined using Western Blot to assess for apoptosis of renal cells. We found that Hem/CLP resulted in an increase in caspase 3 levels and, consistent with the prior trends, PHPS1 injection attenuated the expression of cleaved caspase 3 (Fig. 3d).

### The changes in kidney histopathology induced by Hem/CLP are attenuated by PHPS1 treatment

**Fig. 4:**
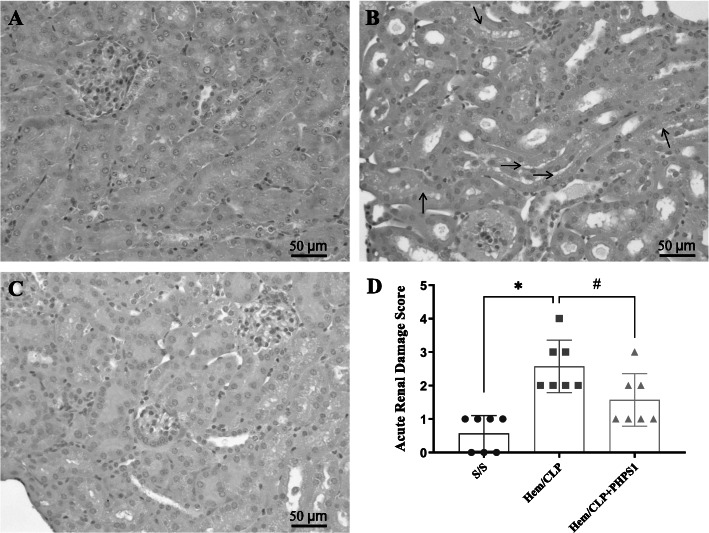
Effects of SHP2 inhibition on kidney histopathologic changes after Hem/CLP. The kidneys were collected 24 h post Hem/CLP or Sham operation. Representative H&E staining of kidney sections for detection of injury by pathological changes. Compared to S/S group (**a**), the kidney from Hem/CLP mice (**b**) exhibited tubular epithelial cell sloughing/detachment (black arrows), edema, inflammatory cell infiltration, loss of brush borders, tubular dilation, and tubular distortion, examined under light microscopy (20X) (scale bar: 50 μm). However, PHPS1 treatment markedly ameliorated these changes and preserved the renal architecture (**c**). The summary data of injury score of 3–5 fields/slide/sample at 200x magnification (**d**). *, *P* < 0.05, versus Sham (S/S); #, *P* < 0.05, PHPS1 treated group (Hem/CLP + PHPS1) versus Hem/CLP + vehicle (Hem/CLP). One-way ANOVA and a Student-Newman-Keuls’ test, Mean ± SD; *n* = 6 mice/group

The histological images we present/examined here where from sections including glomerulus and renal tubules from within the renal cortex. In general, inspection of the kidney suggested that renal cortex was more disturbed by Hem/CLP. As shown in the histology in Fig. 4, Hem/CLP caused an increase in pathological changes including renal tubular epithelial cell sloughing, edema, inflammatory cell infiltration (supplemental data, Figure A; a finding in keeping with our prior observation that Hem/CLP increases kidney myeloperoxidase levels (Biron et al. 2018)), loss of brush borders, tubular dilation, and tubular distortion, when compared to the sham group. However, PHPS1 treatment markedly ameliorated these changes, leading to preservation of the renal architecture.

### The Hem/CLP-induced increase in systemic inflammatory cytokine/chemokine levels is suppressed by PHPS1 administration

**Fig. 5:**
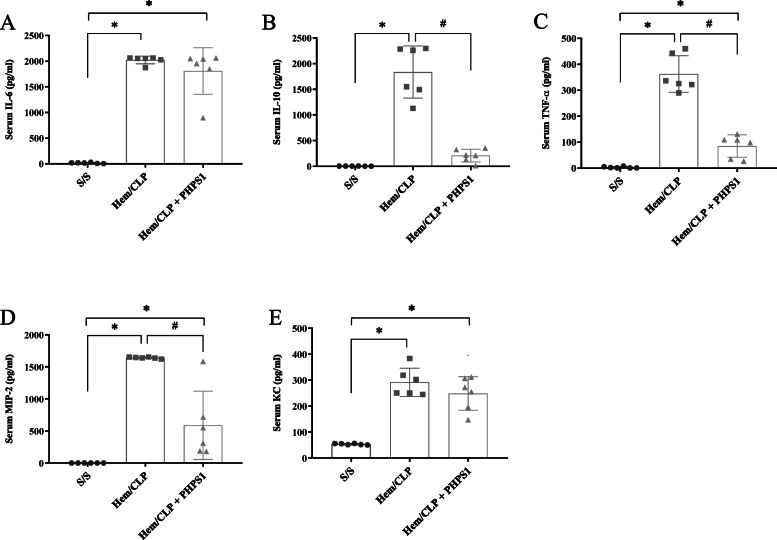
Effects of PHPS1 on systemic plasma levels of inflammatory cytokine/chemokine produced after Hem/CLP. The plasma levels of IL-6 (**a**), IL-10 (**b**), TNF-α (**c**), MIP-2 (**d**) and KC (**e**) in the vehicle or the PHPS1 treated Hem/CLP mice were increased compared to the Sham animals. However, this Hem/CLP-induced increase was markedly suppressed by PHPS1 treatment for IL-10, TNF-α and MIP-2, but not IL-6 and KC levels. *P < 0.05, versus Sham (S/S); # *P* < 0.05, PHPS1 treated group (Hem/CLP + PHPS1) versus Hem/CLP + vehicle (Hem/CLP)*.* One-way ANOVA and a Student-Newman-Keuls’ test, Mean ± SD; *n* = 6 mice/group

The serum levels of inflammatory cytokines including IL-6, TNF-α, and IL-10 as well as chemokines such as keratinocyte chemoattractant (KC) and macrophage inflammatory protein 2 (MIP-2) were significantly increased in the Hem/CLP group when compared with S/S. While administration of PHPS1 dramatically reduced the levels of TNF-α, IL-10, and MIP-2, interestingly, there was no significant change observed in IL-6 and KC levels following Hem/CLP (Fig. 5).

### PHPS1 treatment suppresses the Hem/CLP-induced expression of HMGB1 in kidney

**Fig. 6:**
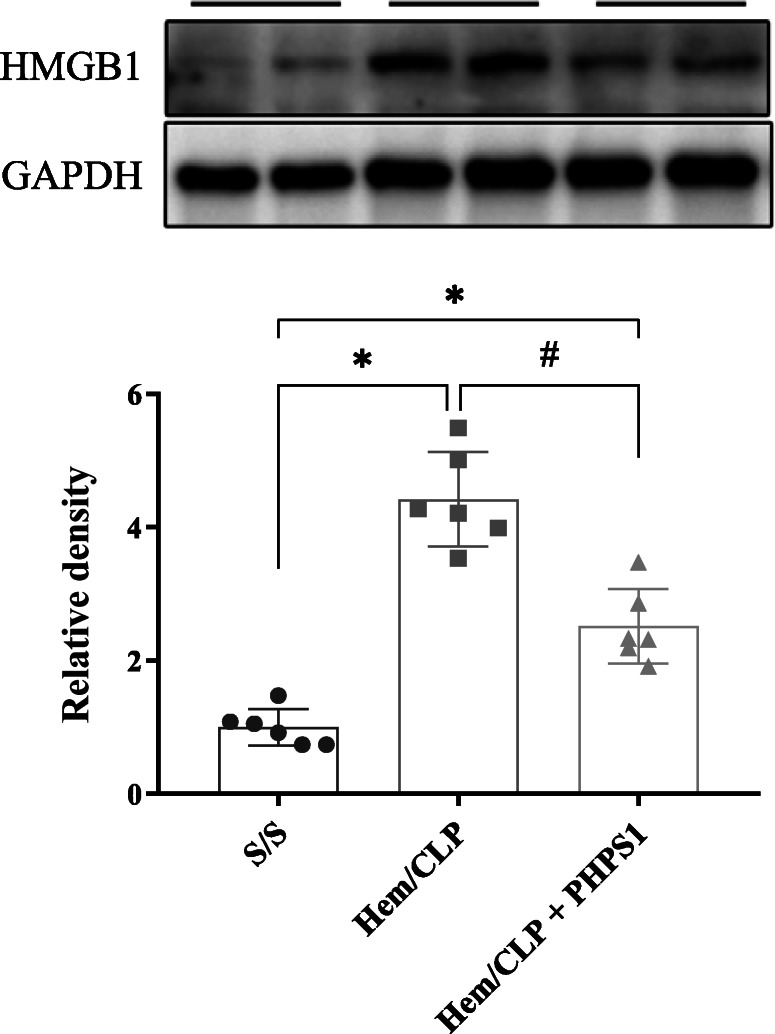
Effects of PHPS1 on expression of HMGB1 in kidney after Hem/CLP. The kidneys were collected 24 h post Hem/CLP or Sham operation and tissue homogenates were obtained. The expression of HMGB1 was determined by western blot. The levels of HMGB1 from Hem/CLP mice was significantly increased compared to Shams. PHPS1 treated Hem/CLP mice showed reduced HMGB1 levels compared to Veh treated HEM/CLP mice. *, *P* < 0.05, versus Sham (S/S); #, *P* < 0.05, PHPS1 treated group (Hem/CLP + PHPS1) versus Hem/CLP + vehicle (Hem/CLP). One-way ANOVA and a Student-Newman-Keuls’ test, Mean ± SD; *n* = 6 mice/group

HMGB1, as a late lethal inflammatory mediator, is reported to play an important role in sepsis outcomes (Vijayakumar et al. 2019; Wang et al. 2001). We observed a significant upregulation of HMGB1 in the renal tissue of Hem/CLP group mice that was also reversed with PHPS1 treatment (Fig. 6).

### PHPS1 inhibits the Hem/CLP-induced activation of the Erk1/2-STAT3 signaling pathway

**Fig. 7:**
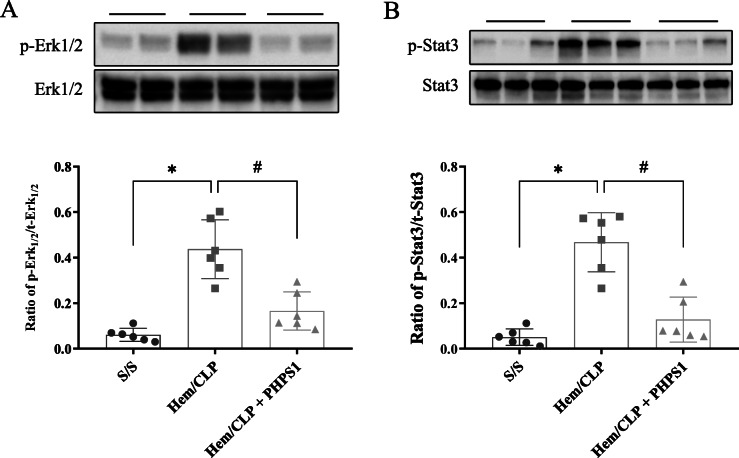
Effects of PHPS1 on expression of activation of Erk1/2 and STAT3 in kidney after Hem/CLP. Twenty four h post Hem/CLP or sham operation, the kidneys were collected and tissue homogenates were obtained. The extent of Erk1/2 (**a**) and STAT3 (**b**) activation (phosphorylation) was determined by western blot. The ratio of phosphorylated Erk1/2 and total Erk1/2 (p-Erk1/2/t-Erk1/2) and phosphorylated STAT-3 and total STAT-3 (p-STAT-3 /t-STAT-3) in kidneys from Hem/CLP mice was significantly increased compared to Shams. PHPS1 treated Hem/CLP mice showed reduced Erk1/2 and STAT3 activation compared to Veh treated HEM/CLP mice. *, *P* < 0.05, versus sham (S/S); #, *P* < 0.05, PHPS1 treated group (Hem/CLP + PHPS1) versus Hem/CLP + vehicle (Hem/CLP). One-way ANOVA and a Student-Newman-Keuls’ test, Mean ± SD; *n* = 6 mice/group

To further illuminate the mechanism through which PHPS1 acts to protect against development of AKI, we sought to determine the extent to which associated pathways including Erk1/2 and STAT3 were activated/phosphorylated in renal tissue. As shown in Fig. 7, Hem/CLP induced not only activation of SHP2 but also Erk1/2 and STAT3, as demonstrated by the marked elevation in the ratios of phosphorylated-SHP2:total-SHP2, phosphorylated-Erk1/2:total-Erk1/2 and phosphorylated-STAT3:total-STAT3. Inhibition of SHP2 activation with PHPS1 treatment markedly reduced this associated rise.

Of note, PHPS1 treatement did not have an effect on S/S mice relative to their basal cytokine levels (supplemental data, Figure B) nor did they elevate NGAL or HMGB1 levels (supplemental data, Figure C1), or activated SHP2 or Erk1/2 (supplemental data, Figure C2).

## Discussion

These findings demonstrate that hemorrhagic shock followed by CLP can induce significant kidney injury in addition to lung injury as we have previously documented (Ayala et al. 2002). Our study also provides some of the first evidence supporting the therapeutic potential of PHPS1 as a post-treatment against AKI induced by shock and sepsis. These findings show that PHPS1 acts to alleviate both systemic and local renal inflammation, as evidenced by significantly diminished serum inflammatory factors and renal tissue expression of HMGB1. These effects may be further mediated through the inhibition of the Erk1/2 and STAT3 signaling pathways.

Many animal models have been used to investigate the development of AKI, including ischemia/reperfusion injury-induced AKI (I/R-AKI), direct bacterial or endotoxin administration and CLP-induced sepsis-associated acute kidney injury (SA-AKI) (Poston and Koyner 2019). However, while these animal models have been useful for ellucidating some of the mechanisms which are believed to underly the development of AKI, they have some limitations. To a certain degree, these models are not in line with clinical pathophysiological processes. In previous studies (Ayala et al. 2002), we did not observe overt evidence of injury to the lung or kidney in response to hemorrhage or CLP model alone. However, once performed in a sequential fashion, hemorrhage followed CLP, indices of lung damage were clearly induced (Ayala et al. 2002) and that appears to be true in the case of AKI based on these findings. In this respect, patients suffering from trauma often have significant blood loss requiring immediate hemostatic interventions and ongoing fluid resuscitation during the course of their recovery. The impact of this initial event on a patient’s capacity to combat subsequent insults, infectious or otherwise, can be significant, even resulting in multiorgan dysfunction and death in the most severe cases. Using a rodent model of hemorrhage with subsequent septic insult in order to approximate aspects of what a traumatic shock patient experiences physiologically, our laboratory has shown that injection of PHPS1, a specific inhibitor of SHP2, attenuates Hem/CLP-induced AKI in mice.

The systemic inflammatory response may be one of the most important pathogenic aspects of this double-hit model. The sharp drop in blood pressure, caused by traumatic shock, is believed to exacerbate hypoperfusion in kidney, inducing primary renal injury, systemic vasodilatation and metabolic derangements (Anderson and Watson 2013; Bonventre and Yang 2011). However, excessive administration of fluids in an attempt to counter hypotension and correct oliguria after AKI, is common but also harmful as it leads to accumulation of fluids within tissues (Boyd et al. 2011; Hjortrup et al. 2016). This may be an instigating factor in the development of multiple organ failure, even if there is no significant source of infection. Therefore, the addition of a secondary septic challenge induces a state of systemic inflammation and anaerobic metabolism. As our data indicates, serum levels of many inflammatory factors including IL-6, TNF-α and IL-10 as well as chemokines KC and MIP-2 were significantly increased in the Hem/CLP group. Alleviation of the systemic and localized kidney inflammation appeared to correspond with a reduction in the extent of renal tissue injury, i.e., decreased renal tissue NGAL levels and kidney pathologic score. Some studies have revealed that HMGB1 plays a role in the pathogenesis of renal disease and have suggested that inhibiting the release of HMGB1 could exert a protective effect against the development of AKI (Ruan et al. 2014). A clinical study showed that patients with AKI had higher serum HMGB1 levels (Zakiyanov et al. 2013) and an animal study of experimental AKI also reported upregulated expression of mouse renal tissue HMGB1 levels (Wu et al. 2010). Our findings also demonstrated increased expression of HMGB1 in mouse renal tissue in the Hem/CLP group, an effect which could be reduced through SHP2 inhibition. Importantly, as HMGB1 is a well documented stimulus for systemic and/or local inflammation (Yang and Tracey 2010; Milić et al. 2017; Gorgulho et al. 2019); its elevation in the kidney would likely be associated with (drive) local tissue cytokine rise as well as contributing to the systemic changes we have documented here. However, this relationship remains to be determined.

The mechanism by which SHP2 inhibition protects against double hit-induced AKI remains poorly understood. We know that SHP2, encoded by the PTPN11, plays a significant role in maintaining homeostasis during inflammation by regulating many facets of cell signaling. Our data indicate that SHP2 inhibition through injection of PHPS1 attenuates the release of inflammatory cytokines and renal tissue HMGB1. Previous studies have revealed that select inflammatory signaling pathways are involved in the processes driving AKI. Teng et al. showed that the lentivirus-mediated silencing of SHP2 improved I/R-induced AKI via inhibition of the TLR4/NF-kB Pathway (Teng et al. 2018). Chen et al. revealed that the PHPS1 exerted a protective effect against atherosclerosis via suppression of the SHP2:ERK pathway activation (Chen et al. 2018). Our current study indicates that post-treatment SHP2 suppression significantly reduced the levels of phosphorylated Erk1/2 and STAT3 in kidney, which is important given that both signaling pathways are widely involved in the inflammatory response.

There are, however, several limitations to our study which warrant discussion. First, while we found that SHP2 blockade reduced systemic and local organ inflammation, the specific cellular targets mediating activation of Erk1/2 or STAT3 signaling remains unclear. In previous studies, our laboratory found SHP1 negatively modulated PD-L1-dependent regulation of Treg cell function during the resolution of shock/sepsis-induced lung injury (Tang et al. 2015). As a component of co-inhibitory receptor signaling, via a number of B-7 checkpoint protein receptor family members, e.g., PD-1, BTLA, etc., SHP1 and/or SHP2 recruitment can serve to alter/suppress T-cell as well as select monocyte/macrophage function(s) in a number of conditions, tumor clearance, viral challenge, wound healing as well as Hem/CLP (Fallon et al. 2018). Thus, it would not be suprising if SHP2 blockade also had effects on such cells and/or functions, especially, if used in a protracted/long-term fashion (not acutely as done here). However, further studies will be needed to confirm the phenotypic changes that occur at a cellular level in leukocytes from Hem alone, CLP alone or combined Hem/CLP mice, as well as if they effect other organs or immune/organ functions.

Secondly, while we chose to use PHPS1 to selectively inhibit SHP2 activation in this study, it has been reported that when SHP2 activation occurs through multiple stimuli simultaneously including IL-1, IL-6 and numerous growth factors (insulin, EGF, PDGF and FGF), they produced a deleterious effect in cancer (Zhang et al. 2015), which is supportive of our findings. Additionally, different species will inevitably have differing sensitivity to such agents, making it unclear whether PHPS1 would necessarily have a similar positive effect in the human trauma patient. In this vein, we feel these findings indicate that further clinical research should be done to establish if such compatibilty issues exist. Also, while we document that pharmacological post-treatment with PHPS1 has an effect on the kidney, and would speculate that it may have a similar action in other organs, like the lung, heart, etc., and might also impact over-all survival; these aspects remain to be examined as components of future studies.

Finally, while both Erk1/2 and STAT3 signaling pathways are known to be involved in mediating IL-6 inflammatory signaling, IL-6 levels in our model were not effected by PHPS1 treatment. This suggests that there may be other cytokines involved in the development of this double hit-induced kidney injury that are mediating the effects of PHPS1 inhibition in the kidney. Though hemorrhagic shock-induced inflammation represents a noninfectious but critical contributory factor in this model, its precise mechanism of action is still unclear. Despite this, our data also indicates that the inhibition of Erk1/2 and/or STAT3 signaling may be a key factor in the protective actions of PHPS1 treatment against Hem/CLP-induced AKI.

## Conclusion

In conclusion, our data support the tenet that SHP2 inhibition attenuates AKI induced by our double-hit model of Hem/CLP and that this protective action may be attributable to its anti-inflammatory property in mitigating activation of the Erk1/2 and STAT3 signaling pathway. We believe this finding may have important potential clinical implications and, thus, warrants further investigation.

## Supplementary information

**Additional file 1: Supplement data, Figure A.** Effects of Hem/CLP on kidney histopathologic changes. The kidneys were collected 24h post Sham-Hem (A) or Hem/CLP (B). Representative H&E staining of kidney sections exhibited tubular epithelial cell sloughing/detachment (black arrows), edema, inflammatory cell infiltration (green arrows), loss of brush borders, tubular dilation, and tubular distortion, when examined by light microscopy (20X) (scale bar: 50 μm). **Supplement data, Figure B.** Effects of PHPS1 on systemic plasma levels of inflammatory cytokine/chemokine in Sham-Hem (SH)/Sham-CLP (SC) control mice. The plasma levels of TNF-α, IL-10, IL-6, and MIP-2 were not detectable (ND) in the PHPS1 or vehicle treated SH/SC mice. Although KC levels were detectable, there was no difference between the 2 sham groups (*p* = 0.5124). Rank Sum Test, Mean ± SD; *n* = 3 mice/group. **Supplement data, Figure C1.** Effects of PHPS1 on expression of NGAL and HMGB1 and in kidney after Sham-Hem (SH)/Sham-CLP (SC). The kidneys were collected and tissue homogenates were obtained 24h post SH/SC. The expression of NGAL and HMGB1 were determined by western blot. The levels of NGAL and HMGB1 were low and no difference between PHPS1 or vehicle treatment SH-SC control mice. Rank Sum Test, Mean ± SD; *n* = 3 mice/group. **Supplement data, Figure C2.** Effects of PHPS1 on the activation of SHP2 and ERK1/2 in kidney after Sham-Hem (SH)/Sham-CLP (SC). The kidneys were collected and tissue homogenates were obtained 24h post SH/SC. The extent of SHP2 and ERK1/2 activation (phosphorylation) was determined by western blot. The ratio of phosphorylated (p) SHP2 and total SHP2 (p-SHP2/t-SHP2) and phosphorylated Erk1/2 (p- Erk1/2 /t- Erk1/2) in kidneys from SH-SC control mice were no difference with or without PHPS1 treatment. Rank Sum Test, Mean ± SD; *n* = 3 mice/group.

## Data Availability

The data supporting the conclusions of this article will be made available by the authors, without undue reservation, to any qualified researcher.

## Abbreviations

AKI: Acute kidney injury
BUN: Blood urea nitrogen
CLP: Cecal ligation and puncture
Cre: Creatinine
ELISA: Enzyme linked immune-assay
EGF: Epidermal growth factor
Erk1/2: Extracellular-regulated kinase
FGF: Fibroblast growth factor
GAPDH: Glyceraldehyde 3-phosphate dehydrogenase
Grb-2: Growth factor receptor-bound protein 2
Gab1: Grb-2–associated binder 1
HE: Hematoxylin and eosin
Hem: Hemorrhage
Hem/CLP: Hemorrhage followed by cecal ligation and puncture mice
HMGB1: High mobility group box 1
ITIM: Intracellular tyrosine inhibitory motif
ITSM: Intracellular tyrosine switch motif
IL-6: Interleukin-6
IL-10: Interleukin-10
I/R-AKI: Ischemia/reperfusion injury-AKI
KC: Keratinocyte chemoattractant
KI: Kidney/body weight index
MIP-2: Macrophage inflammatory protein-2
NGAL: Neutrophil gelatinase-associated lipocalin
NF-kB: Nuclear Factor-kappa beta
PHPS1: Phenylhydrazonopyrazolone sulfonate 1
PTPs: Phosphatase protein tyrosine phosphatase
PI: Phosphatidylinositol
PD-1: Programmed cell death receptor-1
PDL1: Programmed cell death receptor-ligand1
S/S: Sham-Hem followed by sham-CLP mice
SAAKI: Sepsis associated-AKI
SHP2: Src homology 2 domain-containing phosphatase 2
STAT3: Signal transducer and activator of transcription 3
SIRS: Systemic inflammatory response syndrome
Treg: T regulatory lymphocyte
TLR4: Toll-like receptor-4
TNF-α: Tumor necrosis factor
