# Supplementary material for: SHP2 inhibitor PHPS1 ameliorates acute kidney injury by Erk1/2-STAT3 signaling in a combined murine hemorrhage followed by septic challenge model
Source: Mol Med. 2020 Sep 21;26:89. doi: 10.1186/s10020-020-00210-1 (PMC7504828; doi:10.1186/s10020-020-00210-1)

**Supplemental data, Figure A. Kidney Histology**

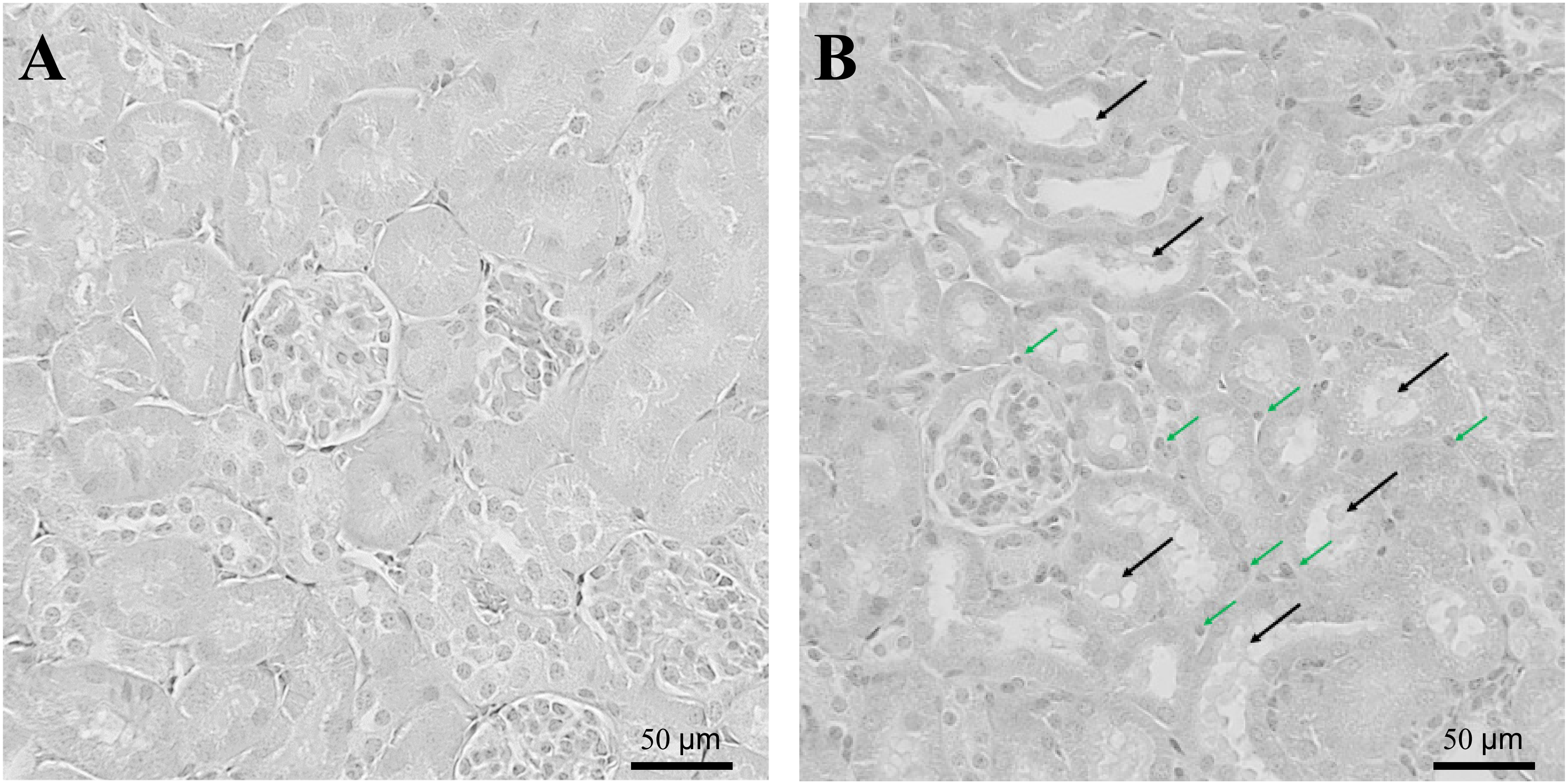

# Supplemental data, Figure B.  Blood Cytokine/Chemokine Levels

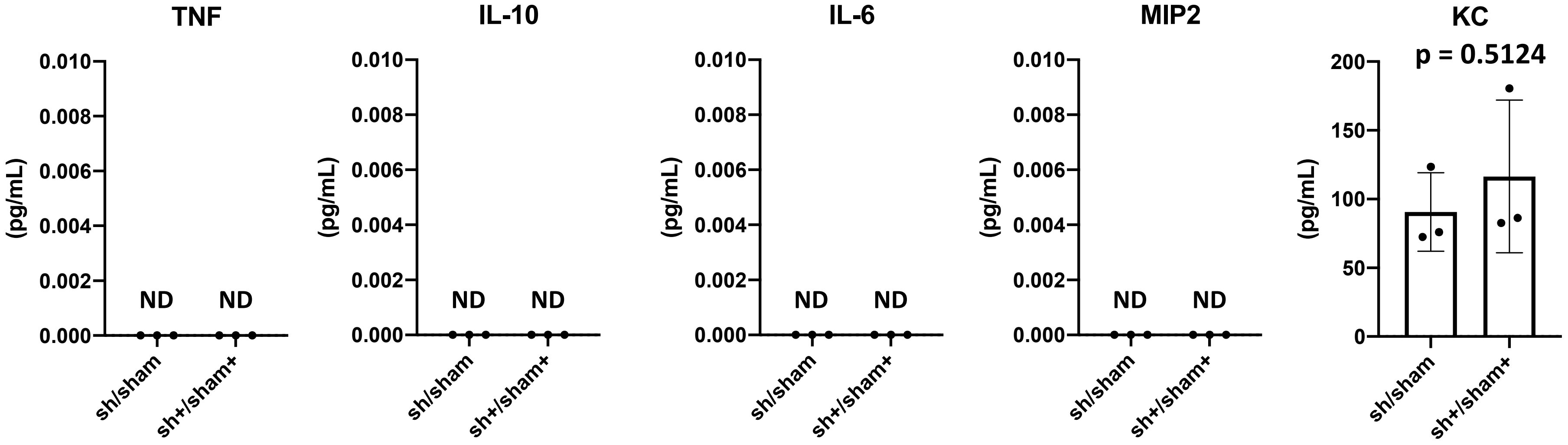

# Supplemental data, Figure C1.  Western Blot (Kidney Tissue)

## NGAL

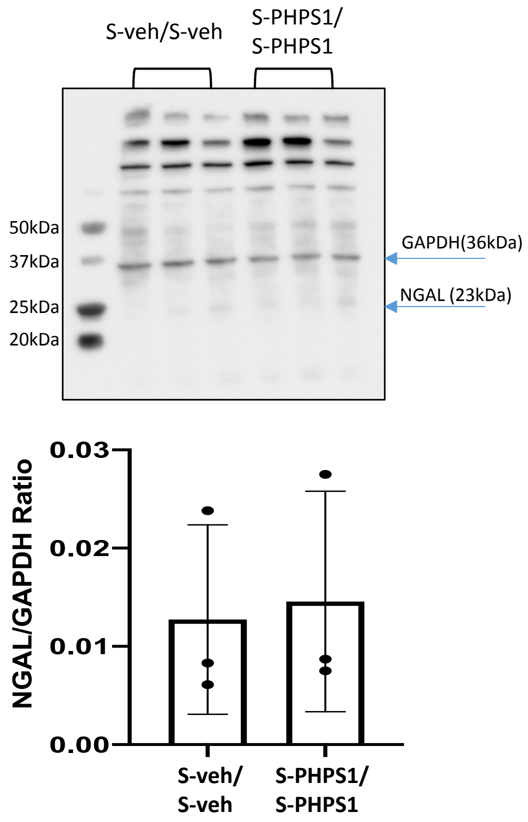


## HMGB1

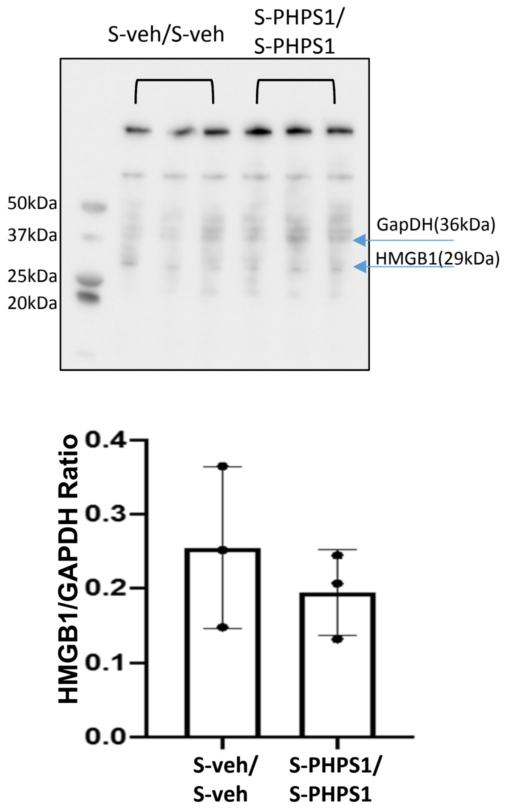

# Supplemental data, Figure C2. Western Blot (Kidney Tissue)

## SHP2

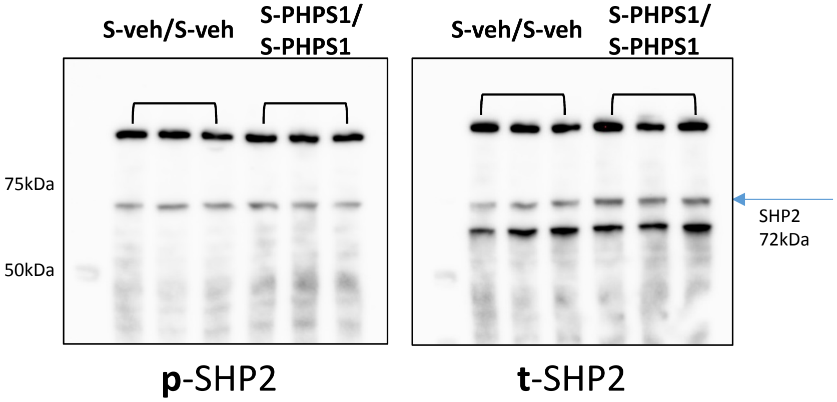


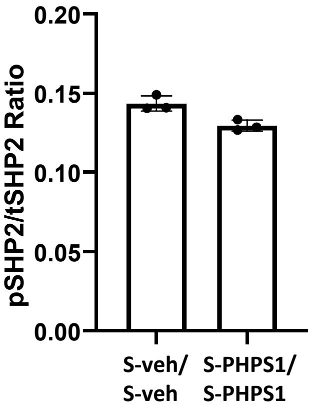


## ERK1/2

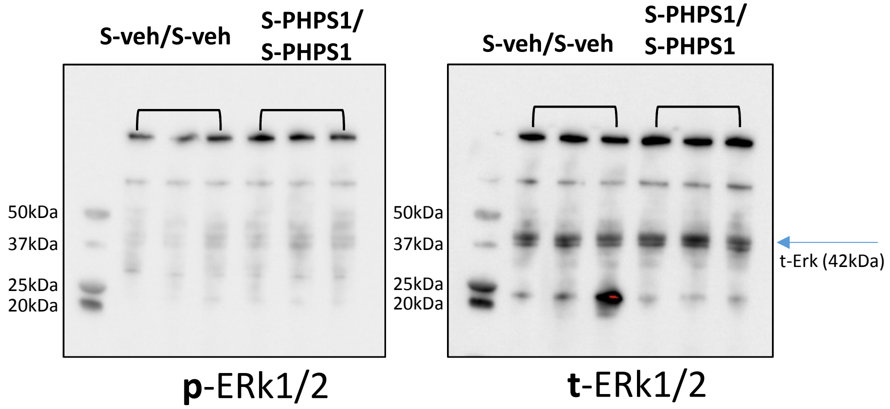


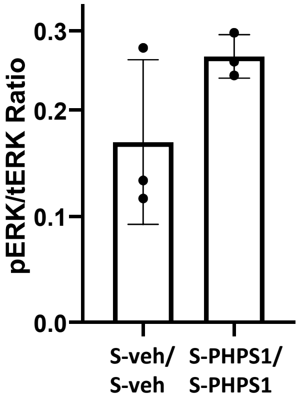

Supplement: Supplementary file 1 — Additional file 1: Supplement data, Figure A. Effects of Hem/CLP on kidney histopathologic changes. The kidneys were collected 24h post Sham-Hem (A) or Hem/CLP (B). Representative H&E staining of kidney sections exhibited tubular epithelial cell sloughing/detachment (black arrows), edema, inflammatory cell infiltration (green arrows), loss of brush borders, tubular dilation, and tubular distortion, when examined by light microscopy (20X) (scale bar: 50 μm). Supplement data, Figure B. Effects of PHPS1 on systemic plasma levels of inflammatory cytokine/chemokine in Sham-Hem (SH)/Sham-CLP (SC) control mice. The plasma levels of TNF-α, IL-10, IL-6, and MIP-2 were not detectable (ND) in the PHPS1 or vehicle treated SH/SC mice. Although KC levels were detectable, there was no difference between the 2 sham groups (p = 0.5124). Rank Sum Test, Mean ± SD; n = 3 mice/group. Supplement data, Figure C1. Effects of PHPS1 on expression of NGAL and HMGB1 and in kidney after Sham-Hem (SH)/Sham-CLP (SC). The kidneys were collected and tissue homogenates were obtained 24h post SH/SC. The expression of NGAL and HMGB1 were determined by western blot. The levels of NGAL and HMGB1 were low and no difference between PHPS1 or vehicle treatment SH-SC control mice. Rank Sum Test, Mean ± SD; n = 3 mice/group. Supplement data, Figure C2. Effects of PHPS1 on the activation of SHP2 and ERK1/2 in kidney after Sham-Hem (SH)/Sham-CLP (SC). The kidneys were collected and tissue homogenates were obtained 24h post SH/SC. The extent of SHP2 and ERK1/2 activation (phosphorylation) was determined by western blot. The ratio of phosphorylated (p) SHP2 and total SHP2 (p-SHP2/t-SHP2) and phosphorylated Erk1/2 (p- Erk1/2 /t- Erk1/2) in kidneys from SH-SC control mice were no difference with or without PHPS1 treatment. Rank Sum Test, Mean ± SD; n = 3 mice/group. [file 10020_2020_210_MOESM1_ESM.pdf]
